# Total arch replacement with frozen elephant trunk technique for Kommerell’s diverticulum with a right-sided aortic arch and aberrant left subclavian artery

**DOI:** 10.1186/s13019-023-02425-9

**Published:** 2023-11-10

**Authors:** Naoto Yabu, Tomoyuki Minami, Ryo Izubuchi, Takahiro Kojima, Ichiya Yamazaki

**Affiliations:** 1 Cardiovascular Surgery, Fujisawa City Hospital, Fujisawa 2-6-1, Fujisawa, Kanagawa 251-8550 Japan; 2 Department of Surgery, Yokohama City University, Fukuura 3-9, Kanazawa-ku, Yokohama, Kanagawa 236-004 Japan

**Keywords:** Right aortic arch, Kommerell’s diverticulum, Aberrant left subclavian artery, Total arch replacement, Frozen elephant trunk technique

## Abstract

**Background:**

Kommerell’s diverticulum with a right-sided aortic arch and aberrant left subclavian artery is uncommon. We perforemed a single-stage procedure with the frozen elephant trunk technique.

**Case presentation:**

A 62-year-old man underwent aortic dissection a year ago, and computerized tomographic angiography performed at that time revealed a right aortic arch, Kommerell’s diverticulum (42 mm), and an aberrant left subclavian artery. We performed one-stage repair through median sternotomy. The cervical branches were exposed during the operation, and a deep hypothermic circulatory arrest with antegrade cerebral perfusion was established. The aorta was transected distally to the origin of the left carotid artery. We inserted a stent graft into the aorta, followed by peripheral anastomosis using a premade 5-branch Dacron graft. The right subclavian artery and the aorta were reconstructed, and the remaining cervical branches were reconstructed after the cross-clamp had been released.

**Conclusions:**

Total arch replacement through median sternotomy was performed for the right aortic arch, Kommerell’s diverticulum, and aberrant left subclavian artery. The frozen elephant trunk technique is allowed to perform a one-stage operation safely.

## Background

Kommerell’s diverticulum with a right-sided aortic arch and aberrant left subclavian artery is uncommon, and surgical indications and standard surgical procedures have not been clearly defined. We report total arch replacement with the frozen elephant trunk technique safely performed for Kommerell’s diverticulum with a right-sided aortic arch and an aberrant left subclavian artery.

## Case presentation

**Fig. 1:**
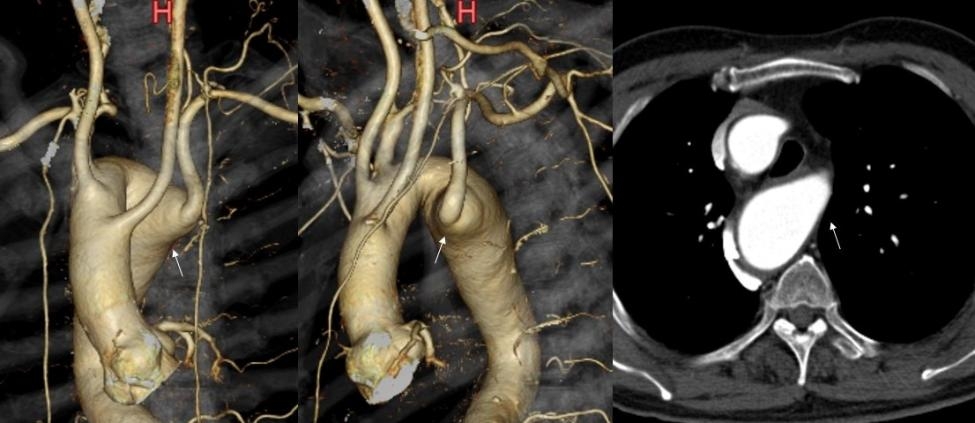
Preoperative computed tomographic angiography revealed a right aortic arch, Kommerell’s diverticulum (arrow), and an aberrant left subclavian artery

A 62-year-old man had a history of acute type B aortic dissection, hypertension, and dyslipidemia. Computed tomographic angiography (CTA) revealed an aberrant left subclavian artery (ALSA) originating from a right aortic arch and Kommerell’s diverticulum (42 mm). Aortic dissection of the thrombotic obstruction type was observed in the descending aorta. He was admitted to the hospital for blood pressure and pain control and was discharged two weeks later. Thereafter, CTA were performed every 3 months, and the dissection cavity almost disappeared 12 months after the onset. The maximum diameter of the descending aorta was 31 mm. The cervical branches originated individually from the arch in the following order: left common carotid artery (LCCA), right common carotid artery (RCCA), right subclavian artery (RSCA), and ALSA (Fig. 1).

The ALSA was located on the dorsal side of the tracheal bifurcation and the esophagus was situated between the trachea and the aorta. and operation of the distal anastomosis was expected to be difficult. Total arch replacement using an open stent graft with a median sternotomy was planned. After induction of general anesthesia, a midline sternotomy was performed, and the left femoral artery was exposed. The cervical branches were exposed before cardiopulmonary bypass (CPB). After heparinization, a 16 Fr cannula was inserted into the left femoral artery; a 20 Fr cannula was inserted into the ascending aorta, which was connected to the arterial line of the CPB circuit. Cannulas were inserted into the superior and inferior vena cava, connected to the venous line of the CPB circuit, and CPB was started. After starting CPB, a vent cannula was inserted through the right upper pulmonary vein, and the whole body was cooled to 27.5 °C with the rectal temperature as an index. Since ventricular fibrillation was observed during cooling, the ascending aorta was clamped, and cardioplegia was administered anterogradely and retrogradely to obtain cardiac arrest. Thereafter, cardioplegia was administered antegrade and retrograde every 20 min. CPB was stopped when the target temperature was attained, and an aortotomy was performed. A cerebral perfusion catheter was placed in each cervical branch, and selective antegrade cerebral perfusion was initiated. As it was difficult to anastomose the distal arch, we assumed the distal part of the LCCA as the anastomosis line and transected the aorta at the same site. 　The RSCA and RCCA were transected, and their proximal sides of RSCA and RCCA were closed with 4 − 0 polypropylene continuous sutures. Each cerebral perfusion catheter was moved distally. A frozen elephant trunk (FROZENIX 35 mm × 90 mm; Japan Life Line, Tokyo, Japan) was anterogradely inserted into the aorta, and a polytetrafluroethylene (PTFE) felt (15 mm) was applied to the outside of the distal aorta. The distal side of the aorta was formed by suturing with PTFE felt, and the aorta and frozen elephant trunk continuously using 4 − 0 polypropylene. A 9-mm Dacron graft (J graft: Japan Life Line, Tokyo, Japan) was previously sutured to a 4-branch Dacron graft (J graft, 26 mm), and the 5-branch Dacron graft was anastomosed to the distal arch using 3 − 0 polypropylene. The lateral branch was connected to the arterial line of the CPB circuit, and lower body perfusion was restarted. Rewarming was started after anastomosis of the RSCA and side branch with 5 − 0 polypropylene. The aorta was transected 10 mm distal to the sinotubular junction. A PTFE felt (10 cm) was applied to the outside of the aorta, and proximal anastomosis was performed with 4 − 0 polypropylene. Terminal warm blood cardioplegia (antegrade and retrograde) was administered, the air was vented, and the cross-clamp was released. The remaining cervical and lateral branches were anastomosed using 5 − 0 polypropylene (RCCA, ALSA, and LCCA, accordingly). The proximal side of the LSCA originating from Kommerell’s diverticulum was closed using a 4 − 0 polypropylene continuous suture. After rewarming, the CPB was withdrawn. The bypass times were as follows: CPB (319 min), SACP (242 min), and lower body circulatory arrest time (86 min with distal perfusion).

**Fig. 2:**
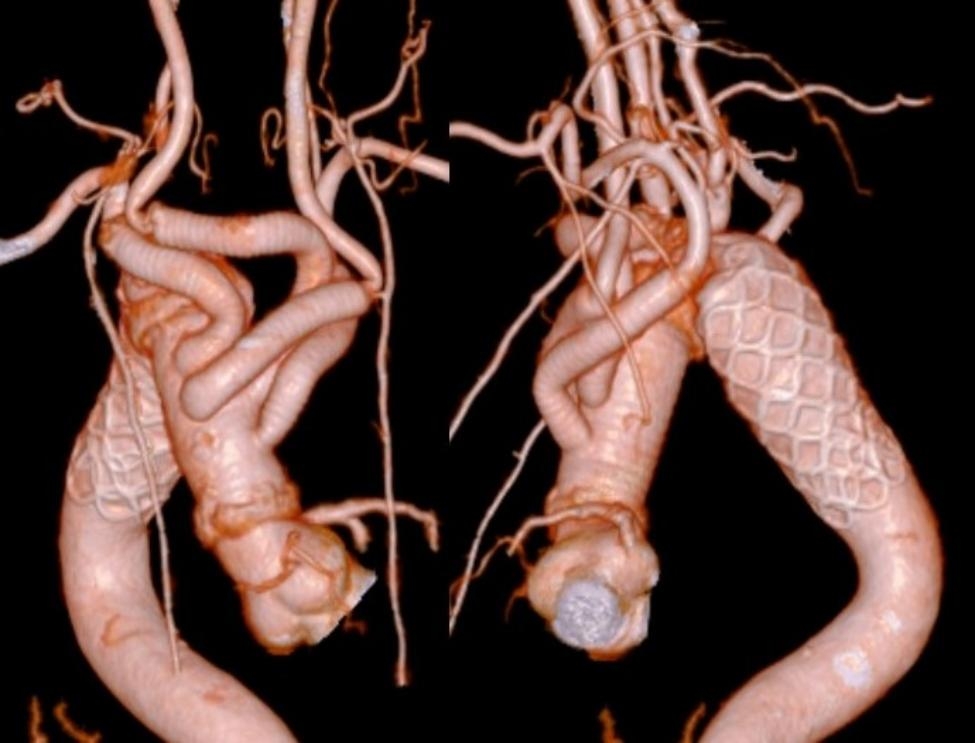
Follow-up computed tomographic angiography at 12 months postoperatively revealed no remaining Kommerell’s diverticulum with no endoleak

After surgery, the patient was admitted to the intensive care unit and extubated 9 days after surgery due to delayed alertness. CT of the head was also performed, but there were no abnormal findings, and the cause of the delayed alertness remains unknown. He was discharged from the hospital 28 days after the operation; however, he needed an extended period of rehabilitation to regain his overall condition due to prolonged intubation caused by the delayed alertness. Follow-up CTA at 12 months postoperatively confirmed no endoleak, anastomotic stenosis, or other problems (Fig. 2).

## Discussion and conclusions

Kommerell’s diverticulum is left as a sac because of incomplete involution of the dorsal aorta during fetal life [1]. The frequency of occurrence of the right-sided aortic arch is approximately 0.1%, and half are associated with an aberrant subclavian artery originating from Kommerell’s diverticulum [2, 3]. Surgical indications for Kommerell’s diverticulum have not been clearly defined. Surgery is performed when symptoms, such as enlargement of the aneurysm, complications of dissection, rupture, compression of other organs, and formation of vascular rings, are observed [4, 5]. It has been reported that the aneurysm diameter is ≥ 50 mm in asymptomatic patients and is indicated for surgery [4]. Conversely, there are reports that patients with a low risk of surgery are indicated for surgery at ≥ 30 mm [5] As this case was complicated by acute aortic dissection and large aneurysm diameter, it was judged to be surgically indicated to prevent rupture. There are two points regarding Kommerell’s diverticulum surgery: resection or closure and ectopic subclavian artery reconstruction. Resection or closure of Kommerell’s diverticulum is often performed with a midline sternotomy or lateral thoracotomy approach [6]. Recently, some reports of treatment with thoracic endovascular aortic repair (TEVAR) have been reported [7]. Although some reports indicate no problem without the ectopic subclavian artery reconstruction, some studies report ischemic symptoms, so reconstruction is considered desirable if possible [8–10]. The typical method of subclavian artery reconstruction is in situ or bypass [4, 5, 10]. Reconstruction in situ portends damage to surrounding organs, such as the esophagus and recurrent laryngeal nerve [11]. Bypass requires a new surgical wound in the neck, and there is a risk of steel and cerebral infarction. In this case, preoperative CT examination showed that the LSCA started from Kommerell’s diverticulum toward the left anterior, and it was expected that surgical operation of the ALSA would be difficult with the right thoracotomy approach. Therefore, we chose a midline approach. However, it was difficult to perform distal anastomosis on the distal side of Kommerell’s diverticulum. Therefore, we chose total arch replacement using a frozen elephant trunk, which can exclude Kommerell’s diverticulum. When using frozen elephant trunks, we leave very little of the graft alone to avoid kinking of the graft portion. Although TEVAR was also an option for this surgical procedure, we decided to select this procedure. Kommerell’s diverticulum was located near the small curvature of the steep aortic arch. Therefore, when TEVAR was performed, there was a possibility of type 1a endoleak due to bird beak. In addition, the use of a larger-than-necessary device to increase the length of the landings would also increase the risk of Stent Induced New Entry. We also considered debranching TEVAR, but the patient’s relatively young age made us think that total arch replacement via median sternotomy would be the best procedure for this patient. The required landing zone was measured by preoperative CTA, and a frozen elephant trunk of 9 cm was selected to avoid spinal cord ischemia. We were careful not to cover up to around Th10-11, where the Adamkiewicz artery generally branches off. The required landing zone was measured by preoperative CTA, and a frozen elephant trunk of 9 cm was selected. Since this disease exhibits different blood vessel running depending on the individual case, it is necessary to carefully examine the CT results before surgery and select the optimal surgical method for each. We used a pre-made 5-branch graft which and a frozen elephant trunk, and safely performed total arch replacement to repair Kommerell’s diverticulum and ALSA via a midline approach.

## Data Availability

Not applicable.
